# Early and Late Luteal Executive Function, Cognitive and Somatic Symptoms, and Emotional Regulation of Women with Premenstrual Dysphoric Disorder

**DOI:** 10.3390/jpm12050819

**Published:** 2022-05-18

**Authors:** Pai-Cheng Lin, Chih-Hung Ko, Ju-Yu Yen

**Affiliations:** 1 Department of Psychiatry, Kaohsiung Municipal Ta-Tung Hospital, Kaohsiung 80145, Taiwan; superfoxcat@yahoo.com.tw; 2 Department of Psychiatry, Kaohsiung Medical University Hospital, Kaohsiung Medical University, Kaohsiung 80756, Taiwan; chihhungko@gmail.com; 3 Department of Psychiatry, Faculty of Medicine, Graduate Institute of Medicine, College of Medicine, Kaohsiung Medical University, Kaohsiung 80708, Taiwan; 4 Department of Psychiatry, Kaohsiung Municipal Siaogang Hospital, Kaohsiung Medical University, Kaohsiung 81267, Taiwan

**Keywords:** PMDD, cognition, executive function, emotional regulation, reappraisal, insomnia

## Abstract

Objective: Cognitive and somatic symptoms were vital factors in developing personalized treatment of depressive disorder. The study aimed to evaluate the following: (1) the cognitive and somatic symptoms of premenstrual dysphoric disorder (PMDD) in the early luteal (EL) and later luteal (LL) phase; and (2) their association with depression and functional impairment of PMDD. Methods: We prospectively evaluated executive function, emotion regulation, cognitive and somatic symptoms, and depression in the EL and LL phases in women with PMDD. Sixty-three women with PMDD and 53 healthy controls completed Simon’s task and questionnaire to assess emotion regulation, inattention, fatigue, insomnia, and depression. Results: Women with PMDD had a poor performance in Simon’s task during the LL phase. They were less likely to exercise cognitive reappraisal during EL and LL phases. Their cognitive reappraisal positively correlated with executive function and negatively associated with depression. In the LL phase, they also experience higher inattention, insomnia, and fatigue, which correlate with the depression and functional impairment of PMDD. Inattention is the most associated factor of PMDD and functional impairment in controlling depression. Conclusion: Executive function was impaired in women with PMDD during the LL phase. Its performance correlated positively with emotion regulation and negatively with depression. The association between inattention and PMDD functional impairment indicates that evaluation and intervention for cognitive impairment were essential in treating women with PMDD. Further studies were required to elucidate the possible etiology underlying these associations.

## 1. Introduction

The Diagnostic and Statistical Manual of Mental Disorders (DSM-5) defines premenstrual dysphoric disorder (PMDD) as a depressive disorder. PMDD is characterized by predictable phasic psychological, cognitive, and somatic symptoms that are aggravated for approximately six days of the late luteal (LL) phase and improve after the onset of menses throughout most women’s reproductive years [1]. Approximately 1% to 8% of women experience PMDD [2,3]. The LL onset of PMDD symptoms suggests the role of fluctuations in ovarian hormones—estrogen or progesterone—in emotional fluctuation [4]. In addition to mood symptoms, cognitive symptoms, such as in-concentration, and somatic symptoms, such as insomnia and fatigue, are the diagnostic criteria for PMDD without comprehensive evaluation [5,6]. Cognitive impairment is a key factor in determining the personalized treatment of depressive disorder [7]. Understanding the role of cognitive function and somatic symptoms in the depression and functional impairment of PMDD may provide insights into developing a personalized intervention.

### 1.1. The Cognitive Function of Women with PMDD

Women with PMDD experience difficulty in attention and cause functional impairment in the luteal phase [6]. Early studies before 2000 in a limited sample-sized demonstrated no significant cognitive functional impairment in attention, working memory, or cognitive flexibility among women with PMDD [8,9,10]. After that, women with PMDD impaired word recall, response inhibition, and working memory in the luteal phase [10,11,12,13,14], demonstrating a higher dorsolateral prefrontal cortex activation during working memory tasks in women with PMDD. These findings consistently indicate deficits in working memory, memory recall, and response inhibition during the luteal phase among women with PMDD. Le et al. (2020) reviewed these studies. They recommended the evaluation of specific cognitive domains, such as executive function, with an adequate sample size in various menstrual cycle phases for use in the diagnosis of PMDD [5]. Most studies evaluating cognition in PMDD have focused on memory, attention, and response inhibition but not on executive function, which refers to higher-order cognitive processes such as monitoring, organizing, flexibility, shifting, and planning. An evaluation of the changes in executive function in the luteal phase might elucidate the cognitive deficit of women with PMDD.

### 1.2. The Somatic Symptoms of Women with PMDD

Sleep problems, including both hypersomnia or insomnia, and fatigue are issues for women with PMDD and are listed as criteria symptoms for PMDD on DSM-5 [1]. Women with PMDD may experience increased sleep-onset latency, may awaken more during asleep, may experience worsened sleep maintenance, and may experience worsened sleep quality during the luteal phase [15]. These sleep disturbances could be explained by the increased progesterone levels and decreased levels of its metabolite (allopregnanolone) during the luteal phase [15]. Sleep disturbance mediates the association between PMDD and depression [16]. This might suggest that sleep disturbance could lead to other complications, such as mood instability [17].

Lethargy, increased fatigability, or a marked lack of energy is another core symptom of PMDD. The symptom severity of fatigue is higher in women with PMDD during the entire menstruation cycle than in those without PMDD [6]. Moreover, fatigue also strongly affects the functional impairment of women with PMDD, especially in the luteal phase [6]. Studies have shown that fatigue may be related to the change in progesterone level during the luteal phase [18]. The onset of these complaints could shed light on the symptoms’ mechanisms in PMDD. However, which phase insomnia and fatigue symptoms that women with PMDD develop have not been adequately evaluated.

### 1.3. The Cognitive Function and Emotional Difficulty in PMDD

Hoyer et al. (2013) emphasized the importance of the interplay of cognition and emotion among women with PMDD [19]. Although no association between mood and cognition was shown in an early study [20], Yen and colleagues [12] demonstrated an association between working memory and irritability. It implies a possible association between cognitive function and mood status in women with PMDD. The association between executive function and emotion regulation [21,22] plays a crucial role in developing depression [23]. Emotion regulation involves modifying emotional response through cognitive reappraisal, particularly under stress [24]. Studies have indicated difficulty in emotion regulation among women with PMDD [25,26]. However, whether cognitive impairment contributes to emotion regulation or depression remains poorly understood. Understanding these associations could contribute to personalized psychological or pharmacological treatment of PMDD.

Thus, this study aimed to investigate the following: (1) the cognitive function, inattention, insomnia, fatigue, and emotional regulation of PMDD in the early luteal (EL) and late luteal phase; (2) the association between inattention, insomnia, fatigue, emotional regulation, and functional impairment of PMDD and the confounding role of depression in these associations; and (3) the association between cognitive function, emotional regulation, and depression of PMDD.

## 2. Methods

### 2.1. Participants

We recruited participants through advertisements at university campuses and on the Professional Technology Temple (PTT), one of the largest online forums in Taiwan. Women with PMDD were included if they met the DSM-5 criteria for PMDD [1]—specifically, there were ≥5 of the listed symptoms in criteria A, with most symptoms being alleviated after the onset of menses. Women who met, at most, one symptom or had two or more mild symptoms with no functional impairment were included as a control group. Individuals currently taking psychotropic, gonadotropic, or hormonal medications, such as contraceptive medication, were excluded.

After obtaining informed consent, 88 and 68 participants were enrolled in the PMDD and control groups, respectively, until no further applicants responded to the advertisement. A psychiatrist then interviewed them to exclude psychotic disorders, bipolar I disorder, and substance-use disorders, except for nicotine dependence, based on the Chinese version of the Mini-International Neuropsychiatric Interview [27]. He also made the diagnosis of PMDD according to the DSM-5 criteria. This resulted in 81 and 68 participants in the PMDD and control groups. After excluding women with irregular menstrual cycles or an unfulfilled symptomatic cycle during testing, as described later, 63 women with PMDD and 53 healthy controls were included in the final analysis. The study sample is the same as another report focusing on eating behavior and leptin [28]. The study was approved by the Institutional Review Board of Kaohsiung Medical University Hospital (KMUHIRB-20130150).

### 2.2. Measures

#### 2.2.1. The Premenstrual Symptoms Screening Tool (PSST)

The tool was developed by Steiner and colleagues [29] and translated categorical DSM-IV criteria into a rating scale with degrees of severity. The PSST contains fourteen 4-point items to assess the severity of PMDD symptoms, from not at all (one point) to severe (4 points), as well as five 4-point items to assess the impairments of function for work, relationships with coworkers and family, social life activity, and home responsibility. The 14 items for symptoms severity have the Cronbach’s alpha of 0.96 and are summed up to represent PMDD severity. The five items for functional impairment have the Cronbach’s alpha of 0.91 and are summed up to represent the functional impairment in this study.

#### 2.2.2. Simon Task with Location-Relevant Trials

According to a previous study, the Simon task was designed by using E prime 3 (Psychological Software Tools) to add a location-relevant trail to the original task [30]. In this task, when the stimulus was a white circle, the response was to be based on its location (right or left); when the stimulus was colored, the response was to be its color—red (left) or blue (right)—regardless of the location of the circle. The task displayed white, red, or blue circles on the right or left 13° from the central horizontal meridian of the screen. The stimuli were displayed for 100 ms after the 500 ms display of the central cue. If the response was incorrect, a message was displayed for a duration of 700 ms and then a duration of 1000 ms after the stimulus. Thus, this test has two incongruent stimuli (red circle appearing on the right side or blue circle appearing on the left side), two congruent stimuli (red circle appearing on the left side or blue circle appearing on the right side), and two location-relevant stimuli (white circle appearing on either side). A block with 60 trials was created pseudo-randomly, containing at least 20 trials of each condition (congruent, incongruent, and location relevant). The participants engaged in four such blocks with an inter-block rest time. A practice block consisting of 12 trials preceded the experimental blocks. We collected six data points: the compatible responses and the reaction times of congruent, incongruent, and location-relevant trials.

#### 2.2.3. Pittsburgh Insomnia Rating Scale, 20-Item Version (PIRS-20)

The tool is a self-report questionnaire derived from the original 65-item of Pittsburgh Insomnia Rating Scale, including 12 items for nighttime and daytime distress symptoms; 4 items for sleep parameters; and 4 items for quality, regularity, and depth of sleep to assess the sleep condition in preceding one week [31,32]. Each item is scored on a 0 (not at all bothered) to 3 (severely bothered) scale, with a total score ranging from 0 to 60, to provide an index of insomnia severity with a cutoff score of 20 for clinical insomnia [32]. The PIRS-20 has a Cronbach’s alpha of 0.95 and has a test–retest reliability of 0.92 [33].

#### 2.2.4. Attention and Performance Self-Assessment Scale (APSA)

The APSA was modified from its original 30-item self-assessment questionnaire, which assesses cognitive impairment in tinnitus [33,34]. It includes two correlated subscales (AP-F1 and AP-F2) defined by nine items, each with a five-point response scale and a recall period of 4 weeks. AP-F1 is dominantly defined by problems with prospective memory problems, whereas AP-F2 is defined by difficulties keeping attention focused [34]. Test–retest reliability of the APS20, AP-F1, and AP-F2 (intra-class correlation coefficients ≥ 0.87) and internal consistency (Cronbach’s alpha ≥ 0.89) are high [34]. We used AP-F1 (prospective everyday memory problems) and AP-F2 (difficulties keeping attention focus) to evaluate for the inattentive symptoms. A higher score indicates a higher deficit.

#### 2.2.5. Fatigue Severity Scale (FSS)

FSS is a 9-item self-rating 7-point (range 1–7) questionnaire that was developed by Krupp and his colleagues to assess fatigue’s severity [35]. The FSS demonstrated a Cronbach α coefficient of 0.93 and differentiated the fatigue between patients with various diseases and healthy subjects [36]. The higher score represents a more severe fatigue one felt subjectively in the past week.

#### 2.2.6. Emotion Regulation Questionnaire

The Emotion Regulation Questionnaire (ERQ) is a 10-item scale designed to measure respondents’ tendency to regulate their emotions in two manners: cognitive reappraisal, assessed using the reappraisal scale (six items); and expressive suppression, assessed using the suppression scale (four items). Respondents answer each item on a 7-point Likert scale, ranging from 1 (strongly disagree) to 7 (strongly agree). The alpha reliabilities were averaged to 0.79 for reappraisal and 0.73 for suppression scales. The test–retest reliability across 3 months was 0.69 for both scales [37].

#### 2.2.7. The Center for Epidemiological Studies’ Depression Scale (CES-D)

The 20-item Mandarin–Chinese version of CES-D [38] is a self-administered evaluation assessing participants’ frequency of depressive symptoms over the last week. The Cronbach’s alpha of CES-D in the present study was 0.78. In this study, it was utilized to evaluate participants’ irritability.

### 2.3. Procedures

#### 2.3.1. Procedures

We requested the mean duration of the menstrual cycle from participants (menstrual-cycle length range 21–35 days with a variation of 7 days or less). Then we traced the last menstruation day. We estimate the predicted onset of the next menstruation based on this information. The early luteal (EL) phase and LL phase were estimated as 9 or 10 days and within three days before the predicted onset of menstruation, respectively. Participants were assessed by Simon’s task and all the above measures in the same session in the morning during EL and LL phases in the same menstrual cycle. Then we traced the exact onset of menstruation. The data were excluded if the menstruation occurred ≥2 days earlier or ≥7 days later than their LL assessment days. In addition to assessments at EL and LL phase in first evaluating menstrual cycle, the PMDD Severity Questionnaire (PMDDQD) [39] was administered once a week for two further menstrual cycles. Symptomatic cycles were defined according to the PMDD criteria of Smith et al.: late-luteal-phase scores were 30% higher than the minimum score during other menstrual phases [40]. Data from participants in the PMDD group that fulfilled the symptomatic cycle criteria for two or more consecutive menstrual cycles were included in the analysis.

#### 2.3.2. Statistical Analysis

We evaluated the age difference (years old), educational level (the highest grades of school), insomnia, inattention, fatigue, emotional regulation, depression, and performance of Simon’s task between the PMDD group and controls by using the independent *t*-test. The repeated measures of ANOVA were utilized to evaluate insomnia, inattention, fatigue, and emotional regulation (dependent variables) as a function of the menstrual-cycle phase, PMDD diagnosis (independent variables), and the interaction terms in control of age and educational level. Pearson’s correlation was used to analyze the correlation between insomnia, inattention, fatigue, emotional regulation, and functional impairment of PMDD. The same analysis also evaluated the correlations between these variables and depression of PMDD. As the performance of Simon’s task was not in a normal distribution (skewness = 2.36, kurtosis = 7.19), the correlations between insomnia, inattention, fatigue, emotional regulation, depression, and Simon’s performance were evaluated by Spearman’s correlation. These multiple comparisons in the correlation analysis were corrected by the Benjamini–Hochberg procedure. Then the forward logistic regression revealed the most critical factors associated with PMDD in control with age and educational level. The linear regression evaluated the most associated factor of functional impairment among the PMDD group. Lastly, the confounding effect of depression was also tested in these two regression analyses. The *p*-values < 0.05 were considered statistically significant for all analyses.

## 3. Results

### 3.1. The Fluctuation of Insomnia, Inattention, Fatigue, and Emotional Regulation within the Luteal Phase

There is no difference in age and educational level between the PMDD and control group in Table 1. The result demonstrates that PMDD women have higher insomnia, prospective everyday memory problems, difficulties keeping attention focused, fatigue, and lower reappraisal in EL and LL phases (Table 1). The repeated measures two factors ANOVA (Table 2) demonstrated the LL exacerbation of insomnia (*F* = 15.19, *p* < 0.001), inattention (*F* = 23.17, *p* < 0.001), and fatigue (*F* = 6.44, *p* = 0.01) among women with PMDD in comparison with the control group.

### 3.2. The Executive Function of PMDD

In the incongruent trials, participants need to resist the proponent effect from location interference. In the LL phase, compared with controls, women with PMDD gave fewer correct responses in incongruent trials (*t* = −2.57, *p* = 0.012), congruent trials (*t* = −2.45, *p* = 0.02), and location-relevant trials (*t* = −2.34, *p* = 0.02) in Table 3. Furthermore, the Simon’s effect (performance in congruent trials minus performance in incongruent trials) was also stronger (*t* = −2.09, *p* = 0.04) in women with PMDD, indicating that they had impaired executive performance in the LL phase compared with controls.

### 3.3. The Association between Somatic Symptoms, Emotional Regulation, Executive Function, Depression, and PMDD Functional Impairment

The Pearson’s correlation (Table 4) revealed that inattention, insomnia, fatigue, and reappraisal significantly correlated with PMDD functional impairment among the PMDD group in the LL phase. They are also correlated with depression. The forward logistic regression demonstrated that inattention is the most associated factor, followed by fatigue, of PMDD in the LL phase (Table 5). In control of depression, inattention was significantly associated with PMDD, but fatigue did not. It suggests that depression could be a confounding factor in the association between fatigue and PMDD. The linear regression for functional impairment within the PMDD group demonstrated the same results. It demonstrated that inattention is a crucial factor that is associated with PMDD functional impairment independent from depression.

Spearman’s correlation demonstrated a significant negative correlation between Simon’s effect and reappraisal and a positive correlation between Simon’s effect and depression.

## 4. Discussion

### 4.1. The Cognitive Function of PMDD

This study is the first to demonstrate that women with PMDD have poor performance in Simon’s task, including incongruent trials, congruent trials, and location-relevant trials, during the LL phase compared with healthy controls. However, no difference was noted during the EL phase. For Simon’s task, the study participants needed to pay attention to their response to congruent trials, be resistant to location interference to give the correct response to incongruent trials, and keep shifting tasks to respond to location-relevant trials. Thus, they required executive effort to complete the task, particularly for incongruent trials. The stronger Simon’s effect of PMDD group during the LL phase, but not during the EL phase, indicated impaired executive function during the LL phase.

Women with PMDD experience a luteal exacerbation of inattention [6]. This study also shows a LL exacerbation of inattention. The follicular phase score in prospective everyday memory problems (14.91 versus 15.90) and difficulties in keeping attention focused (15.20 versus 15.03) in the previous study were similar to those in the EL phase in this presenting study. This result suggested inattention onset at the LL phase. This result aligns with previous studies [11,12,41] confirming cognitive dysfunction during the LL phase.

The logistical regression demonstrated that the inattention was a proximal factor of PMDD relative to depression. It might suggest that cognitive symptoms could have a specific mechanism independent of depression and deserve further evaluation. Furthermore, inattention is the most associated factor of PMDD functional impairment in LL, even in control of depression. It demonstrated the essential role of cognitive symptoms in PMDD. A personalized intervention for cognitive symptoms of PMDD should be developed to attenuate their functional impairment.

### 4.2. The Emotional Regulation of PMDD

Women with PMDD have a low emotion-regulation capacity [25,42,43]. In this presenting study, they are less likely to cope with stress by using cognitive reappraisal during the EL and LL phase. Emotion regulation plays a vital role in managing anxiety across the menstrual cycle [44]. Our result also demonstrated a negative correlation of reappraisal with depression in women with PMDD in the LL phase; thus, supporting the low reappraisal capacity might limit their ability to modify emotional experiences, such as depression. On the other hand, this result could also indicate that PMDD women with depression impaired their reappraisal capacity.

The executive function is associated with emotion regulation [45,46,47], as the ability to regulate emotions, such as through appraisal, could potentially be enhanced through cognitive resources. Furthermore, reappraisal could attenuate the negative response and preserve cognitive resources, thus maintaining adequate executive functioning [45]. This bidirectional interaction might explain the positive correlation between executive function and reappraisal among women with PMDD in this presenting study.

A review by Albert and Newhouse (2019) suggested that estrogen contributes to cognition and emotional function, particularly during stressful events. Thus, a decline in estrogen concentration may explain the mood symptoms in the luteal phase of the menstrual cycle [48]. This might be a pathophysiological mechanism underlying the association between executive function, emotion regulation, and depression in this study. Petersen et al. (2018) demonstrated hypoactivity of the dorsolateral prefrontal cortex, a key region for executive function, among women with PMDD when engaged in emotion regulation [49]. The data imply that impaired executive function might limit the cognitive resources needed to exercise appraisal when processing the symptoms of depression in the LL phase. The role of estrogen in emotional regulation should be evaluated in future studies.

### 4.3. The Fluctuation of Insomnia and Fatigue of PMDD in the Luteal Phase

In the previous study, women with PMDD had higher insomnia and fatigue in the luteal phase than those in follicular phases [6], and this result in line with a previous polysomnographic study in a limited sample [50]. The mean score of PIRS in the follicular phase is 20.96 in the PMDD group [6]. In this study, women with PMDD had a mean score of 23.38 and 29.32 in PIRS at the EL and LL phase, respectively. It might suggest that, at the onset of insomnia symptoms in the EL phase, the severity is mild, but then it is exacerbated severely at the LL phase. However, the follicular score of fatigue (38.77 versus 39.46) in the previous study was similar to that in the EL phase in this presenting study. These presenting results might suggest that fatigue is exacerbated in the LL phase but not in the EL phase. However, these provisional claims are based on data compared in two separate studies. A continuous cross menstrual-cycle investigation was required to prove the EL onset of insomnia symptoms in PMDD. Nevertheless, the early luteal onset of insomnia might indicate that the factors contributing to insomnia could be an early mechanism of PMDD.

Insomnia and fatigue correlated with depression and PMDD functional impairment in the LL phase among women with PMDD in this presenting study. Insomnia and fatigue were all criteria for major depressive disorder [1]. The increased depression reasonably increases insomnia and fatigue. The regression analysis in this study demonstrated that depression confounds the association between fatigue and PMDD and supports this claim. However, the cross-section design could not conclude the causal relationship. The fatigue and sleep disturbance have been suggested to relate to the change in progesterone level during the luteal phase [15,18], which is one candidate mechanism of PMDD [51]. The shared underline factors might also contribute to the association between insomnia, fatigue, and depression.

Nonetheless, several limitations should be considered in interpreting or generating the results in this presenting study. Firstly, the number of subjects is limited in this study because many candidates were excluded according to the criteria for symptomatic cycle [40] in the prospective investigation. Secondarily, the cross-sectional research design could not confirm the causal relationship between cognitive symptoms, somatic symptoms, depression, and PMDD. Thirdly, other potential confounders related to PMDD symptoms, cognitive function, or emotional regulation, such as stress events, had not been controlled in this study. Fourthly, the EL and LL phases were estimated based on the last menstruation period, not on the ovulation test/LH levels. Fifthly, the mean educational level of participants could be high relative to the general population because the participants were recruited from the community around the university in an urban area. Finally, the PMDD symptoms were rated weekly by PMDDSQ, not daily, in the menstrual cycles. Thus, the effect of memory bias or mental averaging could not be prevented.

## 5. Conclusions

Our results demonstrated that women with PMDD impaired their executive function in the LL phase. The impaired executive function correlated to their lower cognitive reappraisal and depression. They experienced the LL exacerbation of inattention and fatigue. Insomnia onset takes place in the EL phase and progresses in the LL phase. Inattention is the most associated factor of PMDD and LL functional impairment with controlling depression. Thus, the cognitive deficit has an essential role in depression and functional impairment of PMDD and deserves future study to elucidate its mechanism for developing effective personalized intervention of PMDD.

## Figures and Tables

**Table 1:** Insomnia, inattention, fatigue, depression, emotional regulation, and symptoms severity in early and late luteal phase among women with PMDD and controls.

Variables	PMDD Group (Mean ± SD) (*n* = 63)	Control Group (Mean ± SD) (*n* = 53)	Independent *t*-Test
Age	25.02 ± 3.51	24.98 ± 3.73	0.052
Educational level	16.38 ± 1.41	16.11 ± 1.15	1.106
PMDD severity	48.54 ± 10.11	27.51 ± 7.44	12.55 ***
Function impairment	10.87 ± 3.11	6.15 ± 1.81	10.176 ***
Insomnia (PIRS)			
Early luteal	23.38 ± 13.28	12.19 ± 8.40	5.508 ***
Late luteal	29.32 ± 13.88	12.15 ± 9.62	7.833 ***
Prospective everyday memory problems			
Early luteal	15.90 ± 6.15	8.66 ± 5.65	6.559 ***
Late luteal	17.66 ± 6.45	7.08 ± 5.06	9.677 ***
Difficulties in keeping attention focused			
Early luteal	15.03 ± 6.71	7.92 ± 5.83	6.027 ***
Late luteal	19.21 ± 7.12	7.38 ± 5.78	9.697 ***
Inattention			
Early luteal	35.46 ± 13.64	18.81 ± 12.21	6.867 ***
Late luteal	41.56 ± 14.21	16.42 ± 11.78	10.224 ***
Fatigue severity scale			
Early luteal	39.46 ± 12.16	27.25 ± 8.04	6.468 ***
Late luteal	46.00 ± 9.21	28.08 ± 11.69	9.236 ***
Depression			
Early luteal	20.97 ± 10.71	8.57 ± 5.69	7.57 ***
Late luteal	29.27 ± 10.69	14.04 ± 6.91	8.92 ***
Reappraisal			
Early Luteal	29.27 ± 5.34	33 ± 5.74	−3.619 ***
Late Luteal	27.48 ± 6.83	32.57 ± 5.46	−4.373 ***
Suppression			
Early Luteal	17.11 ± 3.98	16.34 ± 4.51	0.979
Late Luteal	16.65 ± 4.22	17.87 ± 4.38	−1.522

Note: *** *p* < 0.001. PMDD severity and Functional impairment: total score and subscore of The Premenstrual Symptoms Screening Tool. Insomnia: score of Pittsburgh Insomnia Rating Scale, 20-item version. Inattention: score of Attention and Performance Self-Assessment Scale. Fatigue: score of Fatigue Severity Scale. Depression: score of Center for Epidemiological Studies’ Depression Scale. Reappraisal and suppression: scores on the reappraisal and suppression subscales of the Emotional Regulation Questionnaire.

**Table 2:** The repeated measures ANOVA evaluating insomnia, inattention, fatigue, and emotional regulation of PMDD as a function of group effect and luteal phase effect with control of age and educational level.

		With-Subject Analysis	
	df	Mean Square	*F* (*p*-Value)
Insomnia			
Late Luteal phase	1	33.78	1.11
PMDD	1	10,651.68	43.70 ***
Luteal * PMDD	1	460.41	15.19 ***
Inattention			
Late Luteal phase	1	14.93	0.34
PMDD	1	23,805.29	80.20 ***
Luteal * PMDD	1	1013.83	23.18 ***
Fatigue			
Late Luteal phase	1	0.78	0.01
PMDD	1	12,763.06	79.99 ***
Luteal * PMDD	1	364.93	6.44 *
Reappraisal			
Late Luteal phase	1	1.18	0.07
PMDD	1	1082.11	20.01 ***
Luteal * PMDD	1	34.72	2.20

Note: * *p* < 0.05; *** *p* < 0.001. Insomnia: score of Pittsburgh Insomnia Rating Scale, 20-item version. Inattention: score of Attention and Performance Self-Assessment Scale. Fatigue: score of Fatigue Severity Scale. Reappraisal and suppression: scores on the reappraisal and suppression subscales of the Emotional Regulation Questionnaire. All analyses were in control of age and educational level. The effect of age and educational level were insignificant in all analyses.

**Table 3:** The difference in the performance of Simon’s task between women with PMDD and controls in the early luteal and late luteal phases.

Variables	PMDD Group (Mean ± SD) (*n* = 63)	Control Group (Mean ± SD) (*n* = 53)	Independent *t*-Test
Correct response			
Congruent trials ^a^			
Early Luteal	73.21 ± 3.15	73.51 ± 2.85	−0.539
Late Luteal	73.54 ± 3.39	74.75 ± 1.84	−2.45 *
Incongruent trials ^a^			
Early Luteal	63.68 ± 9.7	65.83 ± 6.65	−1.408
Late Luteal	65.43 ± 11.26	69.66 ± 6.12	−2.567 *
LR correct trials ^a^			
Early Luteal	68.84 ± 6.5	70.49 ± 5.37	−1.472
Late Luteal	72.37 ± 4.88	74.06 ± 2.79	−2.336 *
Reaction time			
Congruent trials ^a^			
Early Luteal	0.4 ± 0.07	0.42 ± 0.07	−1.275
Late Luteal	0.46 ± 0.07	0.46 ± 0.06	0.24
Incongruent trials ^a^			
Early Luteal	0.49 ± 0.07	0.5 ± 0.07	−0.792
Late Luteal	0.39 ± 0.06	0.39 ± 0.06	−0.002
LR trials ^a^			
Early Luteal	0.42 ± 0.08	0.44 ± 0.08	−1.077
Late Luteal	0.39 ± 0.07	0.4 ± 0.07	−0.707
Simon effect ^a^			
Early Luteal	9.52 ± 8.09	7.68 ± 6.23	1.356
Late Luteal	8.11 ± 9.72	5.09 ± 5.55	2.092 *

Note: ^a^ The task performance in Simon’s task—congruent, incongruent, and location relevant trials; * *p* < 0.05.

**Table 4:** The correlation between insomnia, inattention, fatigue, emotional regulation, functional impairment, depression, and executive function in the later luteal phase among the PMDD group.

	Inattention	Insomnia	Fatigue	Reappraisal	Depression
PMDD group					
Functional impairment (r)	0.64 *** #	0.51 *** #	0.62 *** #	−0.26 * #	
Depression (r)	0.68 *** #	0.65 *** #	0.65 *** #	−0.63 *** #	
Simon’s effect (ρ)	0.11	0.06	0.11	−0.28 * #	0.30 * #

Note: r, Pearson correlation coefficient; ρ, Spearman’s rank correlation coefficient; * *p* < 0.05; *** *p* < 0.001; # significant under multiple comparison correction with Benjamini–Hochberg procedure. Functional impairment: subscore of *The Premenstrual Symptoms Screening Tool*. Depression: score of *Center for Epidemiological Studies’ Depression Scale.* Simon’s effect: task performance in Simon’s task. Inattention: score of *Attention and Performance Self-Assessment Scale*. Insomnia: score of *Pittsburgh Insomnia Rating Scale, 20-item version.* Fatigue: score of *Fatigue Severity Scale.* Reappraisal and suppression: scores on the reappraisal and suppression subscales of the *Emotional Regulation Questionnaire.*

**Table 5:** The association between cognitive dysfunction, somatic symptoms, depression, and PMDD in logistic regression among all subjects and in linear regression among the PMDD group in the luteal phase in control of age and education level.

Variables	Wald X^2^	OR	95% CI
			
Model 1: Forward Regress PMDD on insomnia, inattention, fatigue, emotional regulation, and executive function.			
Inattention	14.95 ***	1.137	[1.065, 1.214]
Fatigue	6.30 *	1.089	[1.019, 1.164]
Model 2: Model 1 in control of depression.			
Inattention	10.47 **	1.12	[1.05, 1.20]
Depression	3.30	1.09	[0.99, 1.20]
Fatigue	2.15	1.06	[0.98, 1.14]
	B	Beta	T
Model 3: Forward Regress PMDD functional impairment on insomnia, inattention, fatigue, emotional regulation, and executive function in PMDD group.			
Inattention	0.09	0.41	3.00 **
Fatigue	0.11	0.33	2.27 *
Model 4: Model 3 in control of depression.			
Inattention	0.08	0.38	2.49 *
Fatigue	0.10	0.30	1.95
Depression	0.03	0.09	0.60

Note: * *p* < 0.05, ** *p* < 0.01, and *** *p* < 0.001. Insomnia: Score of *Pittsburgh Insomnia Rating Scale, 20-item version*. Inattention: score of *Attention and Performance Self-Assessment Scale*. Fatigue: score of *Fatigue Severity Scale*. Depression: score of *Center for Epidemiological Studies’ Depression Scale*. Functional impairment: subscore of *The Premenstrual Symptoms Screening Tool*. All analyses were in control age and educational level. The effect of age and educational level were insignificant in all analyses.
